# The Hygiene of Motherhood

**Published:** 1891-02

**Authors:** John Sheppman


### THE HYGIENE OF MOTHERHOOD.

By Dr. John Sheppman.

### PART FOURTH.

In recognizing the close relationship existing between hygiene and motherhood, it certainly becomes necessary for me to enter into a brief description of the scientific and prophylactic methods employed for the prevention of disease. The first and most important element required for the sustenance of life, is air ; how much our health depends upon its purity can readily be understood, when we learn that every individual is compelled by nature to undergo the breathing process about eight hundred and thirty-eight times an hour, and that every breath contains a quantity of food for the blood and the lungs, which, comparatively speaking is much more essential, than the culinary foods for the stomach. The exact proportion, or relative values of different substances can only be estimated by how long a man can exist while totally deprived of their particular nourishment. It has been frequently stated that a man can live three weeks without food ; three days without water ; and three minutes without air. However different our theories may be in regard to the fasting period, it is undoubtedly an authenticated fact that no one can escape death who is confined without air, for a longer time than five consecutive minutes. According to an eminent authority, each human being, in order to maintain perfect health, requires two thousand cubic feet of new air, every hour. If we should compare these figures with the amount which some of us actually breathe, they would not only prove astonishing, but positively appalling.

There is no gift of nature which is more abundantly provided, than air. The rich, and the poor, have equal access to its beneficent influences. It is the elixir of life. It strengthens and invigorates. It is the only natural tonic for the lungs, and through the lungs it purifies the blood, and gives, by its combined forces, a healthy action to the entire circulatory system. It steals through every crack and crevice in order to supply our positive demands, and leaves both health and comfort where its entrance is made welcome. The inestimable value of pure air can only be appreciated by a knowledge of the many ills which are directly traceable to an impoverished condition of stagnant air, which many are in the habit of breathing, and re-breathing, until their constitutions are brought to a delicate state of life uncertainty, when the spirit yearns for a call to that peaceful sleep : "Where grief and pain no longer feast, and sorrow's veil is shorn."

The constant breathing of air which is in the slightest degree impure, has a deleterious action upon our bodies which renders us particularly susceptible to any change of climate, and favors the conditions which are suitable for the propagation of all contagious diseases. Few people exercise sufficient care in this respect, and many habituate themselves to small close apartments, where not a puff of air can enter, and where the sense of smell of the outsider, is almost stifled when the door is opened to admit his presence. No bedroom that contains an unpleasant odor in the morning, is fit to sleep in ; and efforts should at once be made to have it sufficiently ventilated. It is a great mistake to imagine that every current of air brings with it serious colds and sickness; the result is just the opposite in most cases, for, what many really need is nothing else than a daily supply of good fresh air, combined with a moderate amount of outdoor exercise. This advice is applicable to all people, and especially to those who are chronic sufferers of the various pulmonary disorders.

To suppose that the constant inhalation of impure air has a tendency to excite the symptoms of consumption, is not at all unreasonable, when we consider the wonderful mechanism of the lungs, and the efforts which they are continually employing to throw off the poisons of a corrupted air, which has been breathed, again and again, until its foulness has become unbearable, and even worse—overpowering. One drop of the fluid condensed from the air which has come from the lungs has been found sufficiently powerful to cause instant death. Is it any wonder that many are wasting away and slowly dying upon this destructive food? The fear of catching cold, has caused more sickness, than all the *colds* that ever were "caught." With this peevish dread many strip up their doors, bind up their windows, and look upon every puff of wind, as if it were some keen thief, who had come to rob them of their lives.

This custom prevails mostly among the better class, or, "the rich," as they are termed. The poor man, whose comforts are confined to a little frame house, is seldom heard to complain; and his children are always hale and hearty, in spite of the many draughts which come in through the cracks and the rents of every door and window. The infinite value and the immediate necessity of ventilation must not be underrated; for it is this ever changing current that contributes the greatest boon towards the health and happiness of all classes of humanity.

Every apartment should have an inlet for pure air, through a tube from the roof, or, through an opening in the upper sash of the outside window. An outlet of a corresponding size should be made directly opposite. These openings should be made so that they can be regulated according to the state of the weather, but they should never be permitted to be entirely closed. This rule admits of no exceptions, and in cases of sickness it would be better to provide an additional blanket than to deprive the sufferer of the much needed supply of pure oxygen. An open fire-place, in the winter, is considered healthy, with a ventilating aperture near the ceiling in the opposite wall.

The importance of thoroughly airing articles of wearing apparel, must not be forgotten ; and all trunks, closets, and wardrobes should be frequently opened, and their contents exposed to a generous current of outside air. There are many other sources of air pollution, and fewer people would be sick, and there would be much less misery, if some of the simpler precautions would be more closely followed.

The occupants of every room should take into consideration that the amount of air rendered impure by the lungs and the skin of each person has been estimated to be a little over three cubic feet per minute; and that every gas light, or lamp, consumes a quantity of oxygen, and throws off its poisonous carbonic substances, which, when the air is motionless, must be taken into the lungs, and with their sickening influences scattered broadcast through the entire system. Because you cannot smell any thing disagreeable in the air which you breathe, is no safe test for its quality; for, " custom breeds a habit," and habit leads to tolerance, and where tolerance is established, the olfactory nerve has been benumbed, and the atmosphere, although reeking with filth, is no longer offensive to one accustomed to its smell. Becoming habituated to any deleterious odor does not insure immunity from contagious ills, as many are in the habit of believing. If they do not affect the breather's health immediately, that they are not the less injurious. There is no human constitution that will not prematurely sink by continually inhaling a foul and contaminated air. The time of endurance, depends of course upon the strength of the individual, yet no one, of any intelligence, will doubt that diseased conditions will certainly follow a prolonged state of lung starvation.

It must not be forgotten that children breathe considerably more than adults, and on that account especial care should be taken to give them plenty of fresh air. It is an excellent plan to teach children to practice forced inspiration, this exercise performed daily, is an easy and practical way of developing the lungs and the chest.

A marked improvement is at once noticeable in those who having previously existed in small air-tight rooms, timely avail themselves of an abundance of nature's most generous gift, which will not only prevent disease, but effectually overcome feelings of languor, and faintness ; brighten the intellect, and make new creatures of the old wrecks, who, by their very presence, will exalt, ennoble, and glorify the general conditions of mankind.

